# Delirium risk screening and haloperidol prophylaxis program in hip fracture patients is a helpful tool in identifying high-risk patients, but does not reduce the incidence of delirium

**DOI:** 10.1186/1471-2318-11-39

**Published:** 2011-08-11

**Authors:** Anne JH Vochteloo, Sophie Moerman, Boudewijn LS Borger van der Burg, Maarten de Boo, Mark R de Vries, Dieu-Donné Niesten, Wim E Tuinebreijer, Rob GHH Nelissen, Peter Pilot

**Affiliations:** 1 Department of Orthopaedics, Reinier de Graaf Group, Delft, the Netherlands; 2 Department of Surgery, Rijnland Hospital, Leiderdorp, the Netherlands; 3 Department of Psychiatry, Reinier de Graaf Group, Delft, the Netherlands; 4 Department of Surgery, Reinier de Graaf Group, Delft, the Netherlands; 5 Department of Surgery-Traumatology, Erasmus MC, University Medical Center Rotterdam, the Netherlands; 6 Department of Orthopaedics, Leiden University Medical Center, the Netherlands

## Abstract

**Background:**

Delirium in patients with hip fractures lead to higher morbidity and mortality. Prevention in high-risk patients by prescribing low dose haloperidol is currently under investigation.

**Methods:**

This prospective cohort surveillance assessed hip fracture patients for risk of developing a delirium with the Risk Model for Delirium (RD) score. High-risk patients (score ≥ 5 points) were treated with a prophylactic low-dose of haloperidol according to hospital protocol. Primary outcome was delirium incidence. Secondary outcomes were differences between high- and low-risk patients in delirium, length of stay (LOS), return to pre-fracture living situation and mortality. Logistic regression analysis was performed with age, ASA-classification, known dementia, having a partner, type of fracture, institutional residence and psychotropic drug use as possible confounders.

**Results:**

445 hip fracture patients aged 65 years and older were admitted from January 2008 to December 2009. The RD-score was completed in 378 patients, 173 (45.8%) high-risk patients were treated with prophylactic medication. Sensitivity was 71.6%, specificity 63.8% and the negative predictive value (NPV) of a score < 5 was 85.9%.

Delirium incidence (27.0%) was not significantly different compared to 2007 (27.8%) 2006 (23.9%) and 2005 (29.0%) prior to implementation of the RD- protocol.

Logistic regression analysis showed that high-risk patients did have a significant higher delirium incidence (42.2% vs. 14.1%, OR 4.1, CI 2.43-7.02). They were more likely to be residing at an alternative living situation after 3 months (62.3% vs. 17.0%, OR 6.57, CI 3.23-13.37) and less likely to be discharged from hospital before 10 days (34.9% vs. 55.9%, OR 1.63, CI 1.03-2.59). Significant independent risk factors for a delirium were a RD-score ≥ 5 (OR 4.13, CI 2.43-7.02), male gender (OR 1.93, CI 0.99-1.07) and age (OR 1.03, CI 0.99-1.07).

**Conclusions:**

Introducing the delirium prevention protocol did not reduce delirium incidence.

The RD-score did identify patients with a high risk to develop a delirium. This high-risk group had a longer LOS and returned to pre-fracture living situation less often.

The NPV of a score < 5 was high, as it should be for a screening instrument. Concluding, the RD-score is a useful tool to identify patients with poorer outcome.

## Background

Delirium is a common and serious complication in hip fracture patients. It leads to lower functional abilities, longer hospital stay, impaired cognitive function, more admissions to long term special care facilities and higher mortality rates [1-5]. This advocates the importance of preoperative delirium risk assessment.

Reported post-operative incidence rates range widely from 16 to 62% [6]. This broad range can be explained by patient inclusion criteria and different scoring methods for delirium. Furthermore, delirium is frequently undetected or misdiagnosed [7].

Haloperidol is widely used for symptomatic treatment of delirium. However, prophylaxis with haloperidol did not lower delirium incidence, it did reduce duration of episodes and the severity in a recent randomized controlled trial [8].

In 2008 we introduced an integrated hip fracture care pathway that included a Risk Model for Delirium [9]. This model should identify high-risk patients that are subsequently prescribed prophylactic haloperidol. Primary purpose of this surveillance study was to determine whether using prophylaxis would diminish delirium incidence in hip fracture patients. The second aim was to investigate the value of the score and differences between low- and high-risk patients (as determined by the risk model) in delirium incidence, length of stay, return to pre-fracture living situation and mortality.

## Methods

A surveillance was conducted on a series of consecutive admissions for a hip fracture to a 450-bed teaching hospital in Delft, the Netherlands.

### Patients

From January 2008 to December 2009, all consecutive admissions for a hip fracture were registered and prospectively studied with respect to presence of delirium. Thus 529 admissions (522 patients) were recorded. These were all patients with a hip fracture due to a low-energy trauma and of non-pathologic origin. For this study, patients of 65 years and older (445 patients) were included for evaluation. Duration of follow-up was 1 year.

The control group for evaluating the effect of the use of the Risk Model for Delirium (RD-score) was a historical consecutive series of 611 hip fracture patients of 65 years and older admitted between 2005-2007, prior to implementation of our RD protocol.

As this study is an evaluation of our delirium protocol, it is considered to be a "Post Marketing Surveillance". Therefore, approval of a medical ethical committee was not necessary.

### Assessment measures

Uniformed data collection of all patients was achieved by evaluating all patients on admission in a standard procedure and recording, according to our local hip fracture protocol [9]. The following data was collected of all patients; age, gender, having a partner, history of dementia, RD-score, pre-fracture living situation, ASA classification [10], psychotropic drug use, type of fracture, treatment and anaesthesia, in-hospital complications, discharge location, in hospital mortality and length of stay (LOS).

Diagnosis of delirium was based on criteria of the DSM IV [1]. Patients were observed for these criteria by both doctors and nursing staff during their daily rounds and assessments. When signs of delirium were notified, they were recorded in the medical and nursing records. Delirium incidence in this series was scored based on these medical and nursing staff records, directly after discharge. Living situation was assessed at 3 months post-admission by questionnaires sent to all patients. Mortality was assessed until 1 year after hospitalisation, using the digital registration system of the hospital.

Delirium incidence in the historical group (2005-2007) was drawn from our hip fracture database that was built retrospectively by evaluation of the patients' files and complication register.

Assessing the risk for a delirium at admission, using the RD-model (table 1), is a standard part of our local hip fracture protocol [9]. This model was developed in 2004 by the department of Psychiatry in our hospital and uses predisposing risk factors for delirium that were weighted, based on known literature at that moment [11-17]. The model was designed with a cut-off point of 5; patients scoring 5 or more points were considered high-risk patients. For this group delirium prophylaxis is prescribed, being 2 times a day 1 mg of haloperidol. In the case of contra-indications for the use of haloperidol, like Parkinson's disease or Lewy-body dementia, alternative prophylaxis was started. When patients developed a delirium, they were fully assessed to exclude a somatic cause and treated by the psychiatric department. The RD-score and the delirium protocol were implemented fully on the departments of Orthopaedics and Trauma surgery in 2008, as a part of the integrated hip fracture care pathway.

**Table 1:** The Risk Model for Delirium

Predisposing risk factors for delirium	Points
Delirium during previous hospitalization	5
Dementia	5
Clock drawing (displaying 10 past 11)	
- Small mistakes	1
- Big mistakes, unrecognizable or no attempt	2
Age	
- 70 to 85 years	1
- Older than 85 years	2
Impaired hearing (patient is not able to hear speech)	1
Impaired vision (vision less than 40%)	1
Problems in activities of daily live	
- Domestic help or help with meal preparation	0.5
- Help with physical care	0.5
Use of heroin, methadone or morphine	2
Daily consumption of 4 or more alcoholic beverages	2

Total score	

### Outcome

The current cohort was analyzed for differences between low- (< 5) and high-risk (≥ 5) patients for delirium incidence, length of stay (LOS), alternative living situation (ALS) 3 months post-fracture (compared to the pre-fracture situation) and in-hospital, 3- and 12-month mortality.

### Statistical analysis

Categorical data are presented as the number of subjects, along with the percentages. Continuous data are presented as means with standard deviations (SD). The value of the RD-score was evaluated using sensitivity, specificity, the negative predictive value of a low score and the positive predictive value of a high score.

Chi-square test, Fisher's exact test and independent Student's t-test were used as applicable for univariate analysis. A P-value lower than 0.05 was taken as the threshold of significance. LOS was divided in two groups at the level of the median (10 days).

The ability of the RD to discriminate was estimated by the receiver-operating characteristic (ROC) curve.

Univariate analysis was followed by multivariable logistic regression to test the association between the RD and delirium, mortality (in-hospital, 3 and 12-month), LOS, and ALS at 3 months. In these analyses age, gender, ASA score (I/II versus III/IV), psychotropic drug use, institutional residence and known dementia were seen as possible confounders. The analysis regarding return to the pre-fracture living situation was performed on patients that lived independent at home before they broke their hip. To this analysis "having a partner" was added as an extra possible confounder.

The likelihood ratio backward test was conducted to find the best-fit model by selecting the variables one by one. The probability for entry was set at 0.05, the probability for removal at 0.10.

All data were analyzed with SPSS 17.0 (SPSS Inc. Chicago, USA)

## Results

### Patients

In 378 of the 445 patients (85%) the RD-score was completed correctly. A delirium was diagnosed in 102 of them (27.0%). Due to the inability of patients to participate or a patient-to-nurse ratio that was too high at some moments, the RD-score was incomplete or not performed in 67 patients.

These 67 discarded patients, as of an incomplete RD-score, had a delirium incidence of 28.4%, not significantly different from study cohort (P = 0.816). Furthermore, there was no difference in age (82.4 vs. 83.8 years; P = 0.168), nor LOS (15.0 vs. 13.2 days, P = 0.172) and 1-year mortality (35.8% vs. 24.9%, P = 0.061) between the completed RD and non-completed RD-score groups.

### Historical comparison

The mean age of the prospective cohort 2008-2009 (83.7 years) was not significantly different from the historical cohort 2005-2007 (82.9 years) (P = 0.082) The percentage of male patients was 26.2% in the prospective cohort and 24.3% in the historical cohort were the same as well (P = 0.515). No significant differences in delirium incidence were found between the prospective 2008/2009 (27%) and the historical cohorts; 2005 (29.0%, P = 0.28), 2006 (23.9%, P = 0.81) and 2007 (27.8%, P = 0.44). (Chi Square-test)

### RD protocol

The protocol was violated in 49 out of 378 patients (13%); prophylaxis was not started in 26 patients with a score of ≥ 5 and was started in 23 patients scoring < 5.

Delirium incidence in the 23 low-risk patients was 34.8%, significantly higher than in the 182 that were not prescribed prophylaxis, 11.5%. (Pearson Chi-Square, P = 0.003). Delirium incidence in the 26 high-risk patients not started on prophylaxis was 50.0%, not significantly higher than in the 147 that were prescribed prophylaxis, 40.8%. (Pearson Chi-Square, P = 0.38) When the protocol violations were excluded, high-risk patients still had a higher risk of delirium (P < 0.001), a longer LOS (P < 0.001) a higher likelihood of living at an alternative living situation after 3 months (P = 0.001) and higher mortality rates at 3 and 12 months (P < 0.001).

### Value of the RD-score

**Figure 1:**
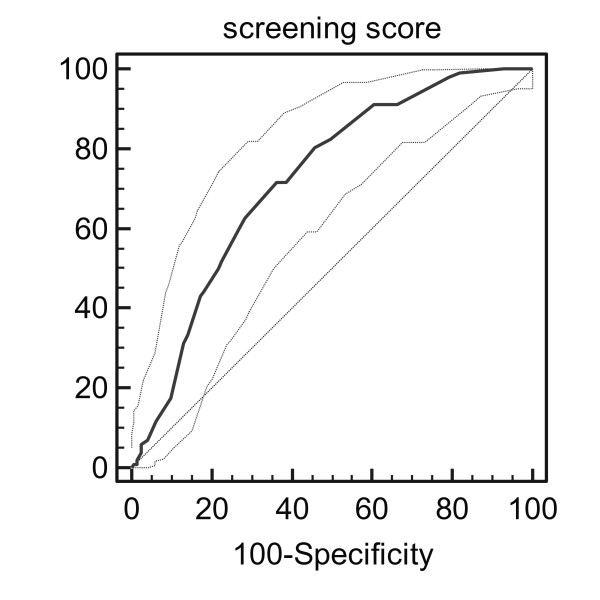
**ROC curve of the RD-score with 95% confidence intervals**. The diagonal indicate results no better than chance.

A receiver-operating characteristic (ROC) curve (Figure 1), made of the continuous outcome of the RD-score showed an area under curve of 0.722 (CI 0.674-0.767, P < 0.0001)The best cut-off point for balancing sensitivity and specificity was 5, corresponding with the pre-study chosen cut-off point.

Sensitivity of the cut-off point was 71.6% (73/102), specificity was 63.8% (176/276). Excluding patients who were not treated according to the protocol, the sensitivity became 74.1% (60/81) and the specificity 64.9% (161/248).

The negative predictive value of a score < 5 (i.e. no delirium) was 85.9% (176/205), the positive predictive value for a score of ≥ 5 (i.e. delirium) was 42.2% (73/173)

### High- and low-risk patients

Specific details of 205 low-risk (score < 5) and 173 high-risk (score of ≥ 5) patients are shown in table 2. High-risk patients were significantly older, more often female, suffering from dementia, ASA classification III-IV, having no partner, residing in an institution, using psychotropic drugs and receiving spinal/epidural anesthesia during surgery.

**Table 2:** Relative risks for different demographic characteristics and outcome parameters with a RD- score ≥ 5 (univariate analysis)

	Score ≥ 5	Score < 5	Relative risk (CI)	P value
	(n = 173)	(n = 205)		
**Age, mean ± SD**	86.6 ± 6.5	81.4 ± 7.1	n/a	< 0.001***
**Female gender**	79.2%	69.3%	1.35 (1.01-1.80)	0.029
**Dementia**	51.4%	0%	3.44 (2.87-4.12)	< 0.001
**ASA -III-IV**	45.7%	22.9%	1.68 (1.36-2.07)	< 0.001
**Institutional residence**	61.8%	10.2%	3.17 (2.54-3.95)	< 0.001
**Having no partner**	79.3%	60.9%	1.74 (1.26-2.41)	< 0.001
**Psychotropic drug use**	51.4%	24.4%	1.82 (1.47-2.25)	< 0.001
**Fracture type**				
**neck of femur**	56.1%	59.0%		0.85*
**(inter) trochanteric**	39.5%	37.0%	0.95 (0.78-1.15)	0.59**
**subtrochanteric**	4.4%	4.0%		
**Treatment**				
**osteosynthesis**	60.5%	50.3%		0.077*
**(hemi-) arthroplasty**	38.5%	46.8%	1.19 (0.98-1.44)	0.072**
**conservative**	1.0%	9%		
**Spinal/epidural anesthesia**	97.5%	91.1%	2.26 (1.05-4.85)	0.006
**Delirium**	42.4%	14.1%	1.98 (1.62-2.41)	< 0.001
**Length of stay ≥ 10 days**	65.1%	44.1%	1.61 (1.27-2.05)	< 0.001
Alternative living situation at 3 months*	62.3%	17.0%	4.25 (2.65-6.80)	< 0.001
**In-hospital mortality**	5.8%	2.0%	1.60 (1.12-2.26)	0.050
**3-month mortality**	23.1%	8.3%	1.69 (1.37-2.10)	< 0.001
**12-month mortality**	37.0%	14.6%	1.77 (1.45-2.17)	< 0.001

* Only calculated for the patients not yet living in institutions (n = 218, n = 32 missing), * Comparing 3 treatment groups; ** RR and p-value comparing femur neck with (inter) trochanteric fractures and osteosynthesis with arthroplasty; *** Independent T-test, CI = confidence interval

At univariate analysis (table 2), patients with a RD-score of ≥ 5 had a higher risk for a delirium, (P < 0.001). Furthermore, they had a longer LOS, a higher chance of living at an alternative living situation after 3 months and a higher 3- and 12-month mortality rate (all P < 0.001).

Multivariable analysis per outcome variable is displayed in table 3. The RD-score was a significantly contributing variable for delirium, length of stay and alternative living situation at 3 months. Age and ASA classification were strong independently contributing variable as well.

**Table 3:** Results of the multivariable logistic regression analysis per outcome variable

Outcome variable	Independent variables	Odds ratio	95% CI	P value
**Delirium**	Screening score ≥ 5	4.13	2.43-7.02	< 0.001
	Age in years	1.03	0.99-1.07	0.082
	Male gender	1.93	1.10-3.39	0.022
**Length of hospital stay ≥ 10 days**	Screening score ≥ 5	1.63	1.03-2.59	0.037
	Age in years	1.06	1.03-1.10	< 0.001
	ASA III-IV	1.55	0.97-2.47	0.069
**Alternative living situation at 3 months**	Screening score ≥ 5	6.57	3.23.-13.37	< 0.001
	Age in years	1.09	1.04-1.06	0.001
**In-hospital mortality**	Age in years	1.14	1.03-1.26	0.014
	ASA III-IV	3.83	1.13-13.0	0.031
	Institutional residence	3.54	0.89-14.0	0.072
**3-month mortality**	Age in years	1.11	1.05-1.17	< 0.001
	ASA III-IV	2.48	1.33-4.61	0.004
	Institutional residence	2.97	1.55-5.68	0.001
**12-month mortality**	Age in years	1.08	1.03-1.12	0.002
	ASA III-IV	2.78	1.60-4.84	< 0.001
	Having no partner	2.22	1.07-4.61	0.033
	Institutional residence	2.06	1.16-3.68	0.014

Female, having a partner, ASA I-II, screening score < 5, not residing in an institution are reference categories

## Discussion

Identification of hip fracture patients at risk for delirium is important in order to start early treatment with medication and psycho-geriatric consultation. Therefore, it is of great value to have an accurate but simple to use, screening instrument.

We used the Risk Model for Delirium (RD-score) to identify patients at risk for delirium and started prophylactic haloperidol in the high-risk group. Large differences between high- and low-risk patients regarding delirium incidence, length of stay, discharge location and mortality were anticipated. However in this study, prophylactic treatment of high-risk patients as identified by our RD-score, did not reduce delirium incidence compared to our historical data. The score did indentify patients with poorer outcome regarding delirium incidence, LOS and return to pre-fracture living situation.

The RD-score had a moderate sensitivity (71.6%) and specificity (63.8%), this is in accordance with other risk models [18]. The negative predictive value (NPV) of a score < 5 was quite high (85.9%), which is very important as a screening instrument should have a high NPV. The consequence of a false positive test (i.e. prophylactic treatment with low-dosis haloperidol in a non-delirious patient) is generally modest as very few side effects of a low dose of haloperidol can be expected. Therefore, its moderate positive predictive value (42.2%) is of lesser importance.

The pre-study chosen cut-off value for the RD-score of 5 was confirmed to be right by the ROC curve analysis. This cut-off point provided a high-risk group with a significant higher relative risk of developing a delirium; OR (adjusted for age and gender) 4.13. Higher age and ASA classification, residing in an institution and absence of a partner suggested a higher vulnerability of the high-risk group. This is demonstrated in outcome; high-risk patients had a longer hospital stay, higher 3- and 12-month mortality, and a higher risk of staying at an alternative living situation at 3 months in univariate analysis. In multivariable analysis, the effect of the RD-score for mortality disappeared.

Several authors described a model that tried to identify high-risk patients for delirium. One study used a cohort of vascular surgery patients [18], another major elective (non-cardiac) surgery patients [15] and 4 others used cardiac surgery cohorts [19-22]. All these models contained items that were not applicable to our patients, while they were patient group specific and designed for an elective surgery population. Kalisvaart et al [8] used a population that contained both elective hip surgery and hip fracture patients. They used visual impairment, disease severity (expressed by the Apache II score) [23], mental impairment (Mini Mental State Examination, MMSE) [24] and dehydration (expressed by blood urea nitrogen/creatinine ratio) as parameters. We chose to develop a simpler model that was easy to use in an acute admission, to achieve maximum use in daily practice. This has been accomplished; 85% of all patients had a complete RD-score. Despite the integration of the RD in a standard patient file, the prophylaxis protocol was violated in 13% of patients. High turnover of doctors in the emergency department may have contributed to these violations.

Older age, cognitive impairment, use of psychotropic drugs (for example benzodiazepines), functional impairment (both in daily activity and clock drawing) visual and hearing impairment were all included parameters that were found to be associated with delirium in a systematic review by Dasgupta et al [25]. Besides these, they found depression, psychopathologic symptoms, psychotropic drugs, institutional residence and medical co-morbidity to be important delirium risk factors. We used institutional residence as a possible confounder in regression analysis, which was of non-significant contribution to risk for a delirium. However, it was a strong predictor of mortality at 3 and 12 months. Psychotropic drug use was associated with a RD-score ≥ 5, but not a predictor of delirium or other outcome in multivariable analysis.

Base on our analysis, adding the factor "male gender" to the RD-score might improve its efficacy as this was a significant contributor to delirium (OR 1.93). This is in contrast to Dasgupta et al [25] who did not find a correlation between male gender and delirium.

Twenty-three low-risk patients were prescribed haloperidol prophylaxis, against the protocol. This group had a higher percentage of delirium than the rest of the low-risk group, which was not hypothesized. The doctor that prescribed haloperidol against protocol might be triggered by patient factors that are not taken into consideration by the score but that do predispose to a delirium as they have a higher delirium incidence.

The prospective character of the study, its large sample size and the use of a predefined risk-stratification model are important issues for interpretation of our results. The main limitations are the subjectivity of determining a delirium and mental impairment of a patient. In our study, delirium was diagnosed based on clinical examination, as stated in the DSM IV [1]. We did not use a measuring instrument like a Confusion Assessment Method [7] to establish delirium. A second limitation was, that in cognitively impaired patients it is difficult to distinguish between delirium and cognitive impairment. Furthermore, patients were scored for known dementia based on history taking and information from digital patient files, a cognitive impairment score like the MMSE was not used [24]. Another limitation is the comparison of the delirium incidence in the whole cohort with the historical cohort. Ideally, we would have compared only the high-risk groups of both cohorts. However, we could not identify high-risk patients in the historical group as the RD-score was implemented fully in 2008. We did demonstrate that both cohorts were comparable regarding mean age and number of male patients, being the main risk factors in the multivariable analysis of the prospective cohort, besides a high RD-score. Therewith one could have observed a decline in delirium incidence due to prophylaxis with haloperidol.

Haloperidol is widely used for symptomatic treatment of delirium, as prophylaxis it has a more disputable reputation. In one small study in gastrointestinal surgery patients, haloperidol prophylaxis was proved effective in reducing delirium incidence [26]. However, a large study in hip fracture patients [8] did not support this finding. Our protocol was developed with the intention to reduce delirium incidence by earlier identification of patients at risk with an objective scoring system, the RD-score. Compared to our historical data, however, we saw no decline in delirium incidence. This corresponded with a recent Cochrane review [27] on interventions preventing delirium. It stated that pro-active geriatric consultation could reduce delirium incidence, but that low-dose haloperidol prophylaxis did not diminish delirium rates [27]. Kalisvaart et al. [8] showed that low-dose haloperidol prophylaxis can reduce severity and duration of delirium and that this may shorten LOS. During the study period, we started using the Delirium Observation Scale [28] to monitor depth and duration of a delirium. However, this instrument was not yet used in a consistent way over the study period to take these data in account for this analysis. Further research should focus more on depth and duration of delirium instead of incidence, since this might give better inside in efficacy of prophylactic treatment. We believe that more emphasis should be given on non-pharmalogical interventions to prevent a delirium. These interventions include providing orientation with calendars, clocks and photographs and maintain day-night rhythm. However, they take valuable manpower from the nursing staff. When these interventions can be targeted to the high-risk group (as identified with the RD-score) it would be preferable.

## Conclusions

Prescribing Profylactic haloperidol to high-risk patients as identified by the Risk Model for Delirium did not reduce delirium incidence in a cohort of hip fracture patients.

The RD-score did prove to be an accurate tool for indentifying high-risk patients with poorer outcome regarding delirium incidence, length of stay and return to pre-fracture living situation.

## Competing interests

The authors declare that they have no competing interests. None of them have received any fundings regarding this study or the preparation of the manuscript.

## Authors' contributions

AV, SM, MB. BB, WT and PP participated in the design of the surveillance. AV, SM, and BB wrote the article. AV, DN and MV were responsible for data collection. WT performed the statistical analysis. PP and RN reviewed the article and supervised the surveillance and analysis. All authors have read and corrected draft versions of the manuscript and have approved the final manuscript.

## Pre-publication history

The pre-publication history for this paper can be accessed here:

http://www.biomedcentral.com/1471-2318/11/39/prepub
